# Deep learning-based malaria parasite detection: convolutional neural networks model for accurate species identification of *Plasmodium falciparum* and *Plasmodium vivax*

**DOI:** 10.1038/s41598-025-87979-5

**Published:** 2025-01-30

**Authors:** Diego A. Ramos-Briceño, Alessandro Flammia-D’Aleo, Gerardo Fernández-López, Fhabián S. Carrión-Nessi, David A. Forero-Peña

**Affiliations:** 1 School of Systems Engineering, Faculty of Engineering, Universidad Metropolitana de Caracas, Caracas, Venezuela; 2 Biomedical Research and Therapeutic Vaccines Institute, Ciudad Bolívar, Venezuela; 3 “Luis Razetti” School of Medicine, Universidad Central de Venezuela, Caracas, Venezuela; 4 Department of Electronics and Circuits, Faculty of Engineering, Universidad Simón Bolívar, Caracas, Venezuela; 5 Immunogenetics Section, Laboratory of Pathophysiology, Centro de Medicina Experimental “Miguel Layrisse”, Instituto Venezolano de Investigaciones Científicas, Altos de Pipe, Venezuela; 6 Department of Infectious Diseases, Hospital Universitario de Caracas, Caracas, Venezuela

**Keywords:** Malaria, *Plasmodium* infection, Artificial intelligence, Deep learning, Neural network model, Medical image processing, Malaria, Image processing, Machine learning, Data processing, Computational models

## Abstract

Accurate malaria diagnosis with precise identification of *Plasmodium* species is crucial for an effective treatment. While microscopy is still the gold standard in malaria diagnosis, it relies heavily on trained personnel. Artificial intelligence (AI) advances, particularly convolutional neural networks (CNNs), have significantly improved diagnostic capabilities and accuracy by enabling the automated analysis of medical images. Previous models efficiently detected malaria parasites in red blood cells but had difficulty differentiating between species. We propose a CNN-based model for classifying cells infected by *P. falciparum*, *P. vivax*, and uninfected white blood cells from thick blood smears. Our best-performing model utilizes a seven-channel input and correctly predicted 12,876 out of 12,954 cases. We also generated a cross-validation confusion matrix that showed the results of five iterations, achieving 63,654 out of 64,126 true predictions. The model’s accuracy reached 99.51%, a precision of 99.26%, a recall of 99.26%, a specificity of 99.63%, an F1 score of 99.26%, and a loss of 2.3%. We are now developing a system based on real-world quality images to create a comprehensive detection tool for remote regions where trained microscopists are unavailable.

## Introduction

Malaria, an infectious disease caused by *Plasmodium* parasites, remains a significant public health problem, particularly in low- and middle-income countries^1^. In 2023, there were an estimated 263 million malaria cases across 83 malaria-endemic countries, resulting in approximately 597,000 deaths. Africa had the highest infection rate, with 246 million cases, followed by the Eastern Mediterranean region and Southeast Asia with 10 million and four million cases, respectively^2^. Accurate malaria diagnosis is crucial for effective treatment, as it involves correctly identifying the *Plasmodium* species, given that treatment varies between species. Microscopy is the gold standard for malaria detection^3–6^. This method involves two tests: thick smear and thin smear. In a thick smear, the presence of malaria is confirmed by identifying *Plasmodium* parasites, while a thin smear detects the specific parasite species infecting the patient^4–6^. Despite being the gold standard, traditional microscopy faces two major obstacles as it is operator-dependent: the chance of human error and a declining number of trained personnel capable of making accurate diagnoses^7^. These challenges have paved the way for emerging diagnostic alternatives based on artificial intelligence (AI), which offer promising solutions to overcome the limitations of traditional microscopy^8–13^.

Innovations in AI have improved our diagnostic capabilities, allowing the medical community to better interpret complex data such as images^14^. One key area of AI is machine learning (ML), which uses training algorithms to learn from historical data and make accurate predictions^15,16^. Within ML exists deep learning (DL), which is a subset that utilizes neural networks with multiple layers to detect patterns in data^17^. These technologies have been assessed through numerous medical fields, including the diagnosis of infectious diseases^18^. The use of convolutional neural networks (CNNs), a DL method mainly used for image processing, allows the development of tools capable of analyzing and interpreting medical images with high accuracy^19–21^. CNNs learn by applying convolutional filters to base images, extracting relevant features such as edges, textures, and shapes. These features pass through the multiple layers of the network, where each layer learns more detailed representations of the image^22,23^. This hierarchical learning process enables CNNs to recognize patterns and make accurate predictions regarding the presence of infectious microorganisms, such as bacteria and parasites, in various sample types, including blood. In the context of malaria, utilizing these technologies may be especially beneficial in regions with limited resources, where traditional diagnostic methods often face significant limitations. Previous models have been efficient in detecting malaria parasites from red blood cell (RBC) images. However, many still face limitations when it comes to differentiating between species^8–12,24^ and the few that have addressed this challenge are not yet close to achieve an optimal performance^25^.

Developing an AI model that accurately identifies *Plasmodium* species not only improves malaria diagnosis but also helps deliver targeted treatments, ultimately enhancing patient outcomes and reducing the overall disease burden. The CNN-based model for multiclass classification of malaria-infected cells developed in our study bridges these gaps and accurately distinguishes *P. falciparum*, *P. vivax*, and uninfected white blood cells. Unlike previous methods that analyze entire microscopic fields^26^, our model focuses on individual cells within a region of interest (ROI), enhancing overall detection precision. This targeted approach allows for more accurate detection and classification of malaria parasites on thick smear images, providing a valuable second opinion for microscopists who need to verify suspicious cells.

## Results and discussion

The experiments were conducted on a system with Windows 10 64-bit, equipped with an Intel Core i7-10700K CPU, 930 GB SSD, 32 GB of RAM, and an Nvidia GeForce RTX 3060 GPU. We used a dataset of 5,941 thick blood smear images (microscope level) from the Chittagong Medical College Hospital, which was processed to obtain 190,399 individually labeled images (cellular level)^27^. We integrated preprocessing techniques into our CNN model, which includes up to 10 principal layers that enhance its performance. Using fine-tuning techniques, such as residual connections and dropout, we improved the model’s stability and accuracy. We also used a batch size of 256, 20 epochs, a learning rate of 0.0005, the Adam optimizer, and a cross-entropy loss function. Data was split into 80% for training, 10% for validation, and 10% for testing. The model’s performance was evaluated using metrics such as accuracy, precision, recall, specificity, F1 score, confusion matrices, and train vs. validation loss graphs. A variation of the K-fold method was employed to robustly assess the model’s generalization capacity^28^.

### Model performance based on applied image processing techniques

Table 1 shows the performance of the proposed DL model after applying various image preprocessing techniques with an 80:10:10 split for training, testing, and validation, respectively^29^. This data distribution follows expert guidelines to maximize the effectiveness of the model’s training and evaluation. The percentage allocated to validation is crucial for fine-tuning parameters, while the test set provides a reliable measure of the model’s performance against unseen data. In Table 1, best performance values are highlighted in bold. This was achieved by the model that included a seven-channel input tensor, demonstrating excellent results in detecting parasites, with an accuracy of 0.9961, precision of 0.9942, recall of 0.9942, specificity of 0.9971, F1 score of 0.9942, and loss of 0.0225. The performance of these results progressively improved as the proposed image preprocessing techniques were added, including the enhancement of hidden features and the application of the Canny Algorithm to enhanced RGB channels. This shows that adding more channels, which allow for extracting richer features, further boosts the model’s performance, underscoring the value of incorporating advanced image preprocessing techniques.

**Table 1:** Performance metrics of the proposed model under different applied preprocessing techniques.

Image preprocessing techniques applied to the proposed model	Accuracy	Precision	Recall	Specificity	F1 score	Loss
Model without image preprocessing or Canny algorithm (three-channel input tensor)	99.20	98.8	98.8	99.4	98.8	4.65
Model with image preprocessing, without Canny algorithm (six-channel input tensor)	99.41	99.11	99.11	99.56	99.11	3.32
**Model with image preprocessing and ** **Canny algorithm (seven-channel input tensor)**	**99.61**	**99.42**	**99.42**	**99.71**	**99.42**	**2.25**
**Model with image preprocessing**,** Canny algorithm ** **(seven-channel input tensor) and cross validation**	**99.51**	**99.26**	**99.26**	**99.63**	**99.26**	**2.3**

### K-fold cross validation results

**Fig. 1:**
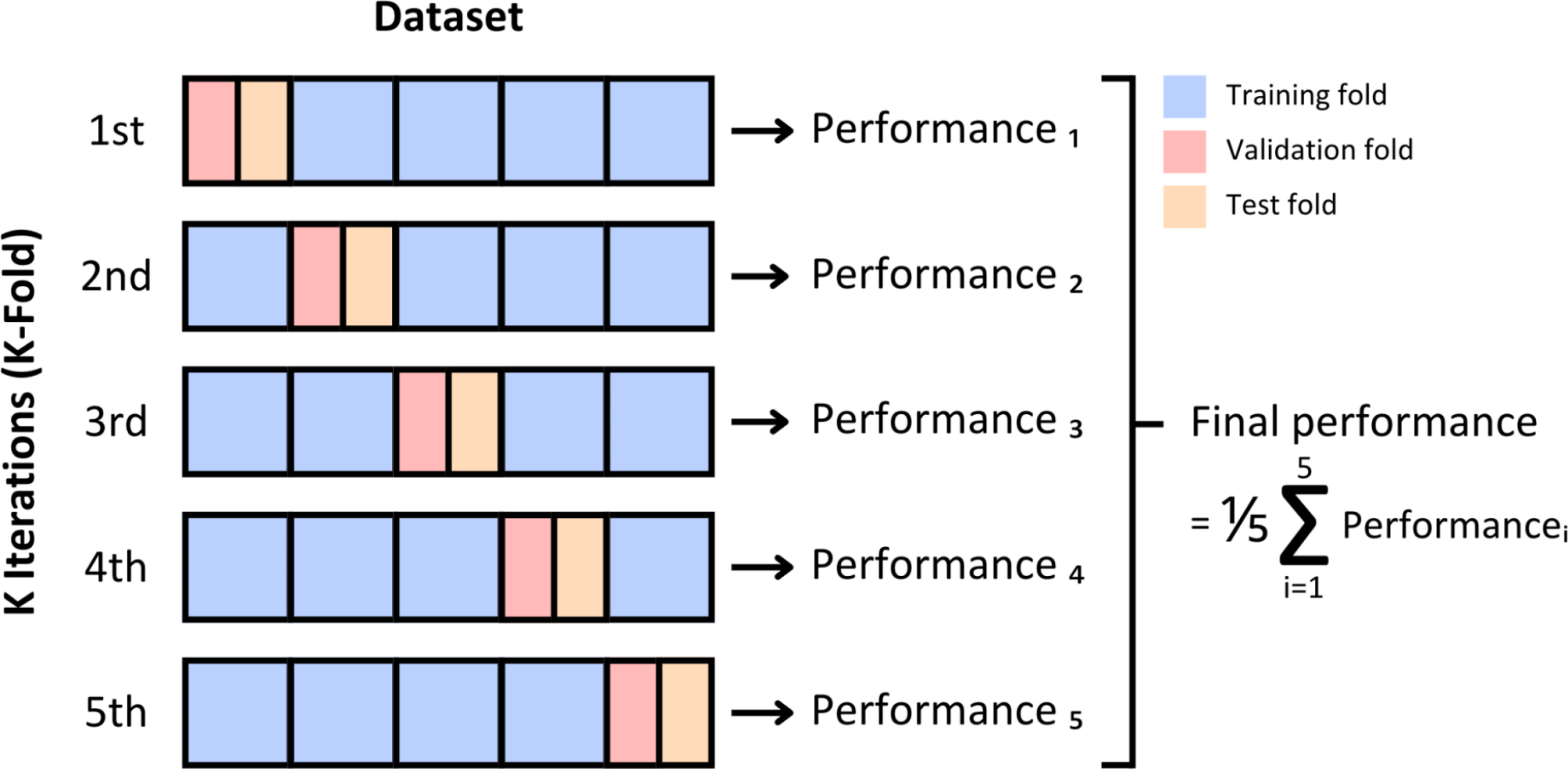
Variant K-Fold cross-validation process applied in this study (Caracas, 2024).

Cross-validation is a key methodology in ML, used to evaluate a model’s ability to generalize unseen data and provide a robust estimate of its performance. Among the various techniques within cross-validation, K-fold is particularly well-suited for assessing a model’s robustness across classification, detection, and other complex tasks^28,30^. In this study, we implemented a specific variant of the K-fold method, dividing the dataset into five equally sized folds using the StratifiedKFold class from the scikit-learn library. In each iteration, four folds were used for training, while the remaining fold was split equally for validation and testing. After completing the five iterations, the results were averaged to obtain the model’s overall performance metrics (Fig. 1). The proposed model achieved an accuracy of 0.9951, precision of 0.9926, recall of 0.9926, specificity of 0.9963, and F1 score of 0.9926.

### Loss function

**Fig. 2:**
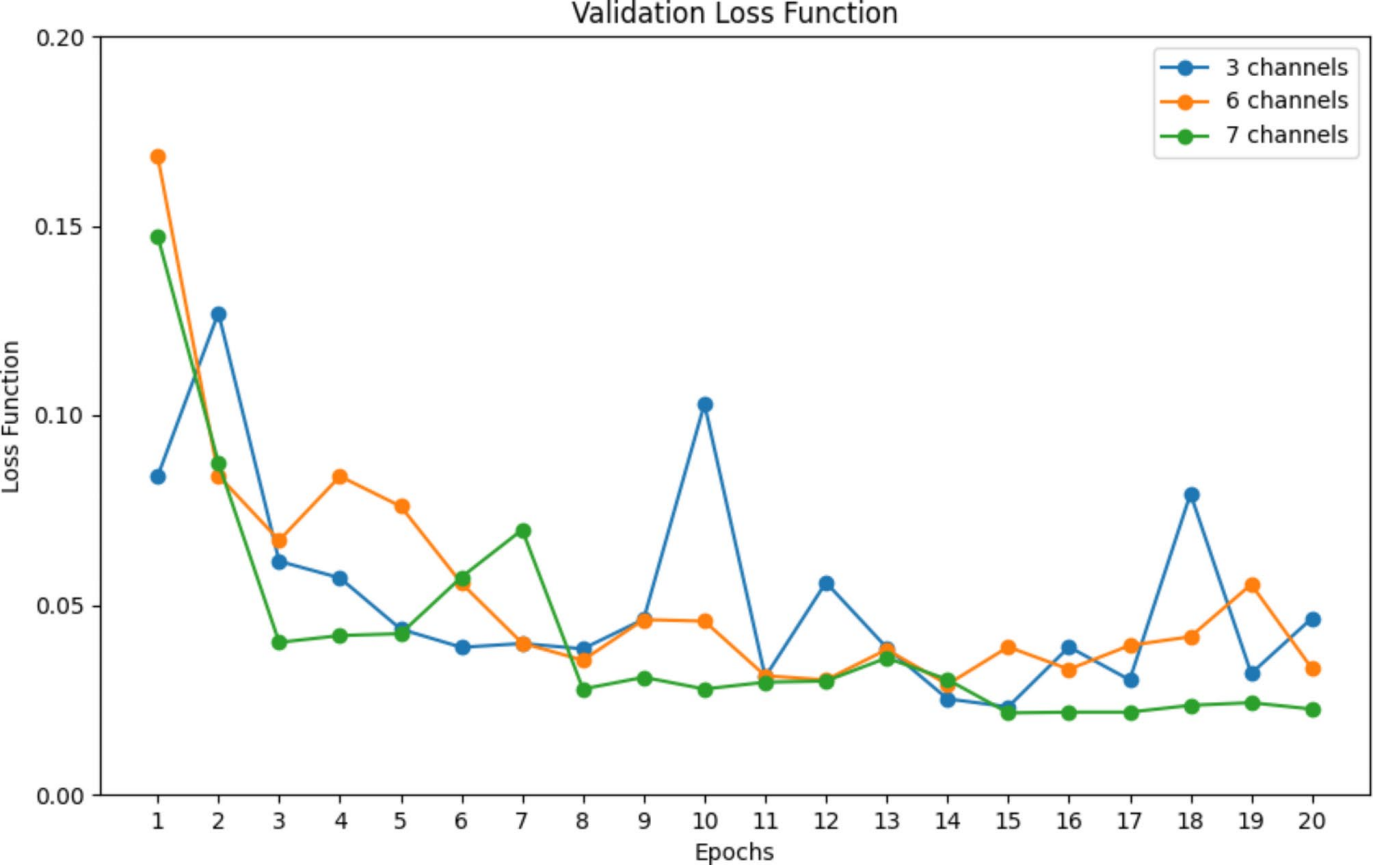
Validation loss results for proposed models by tensor input channels (Caracas, 2024).

The training and validation loss functions are key indicators of model performance, revealing the model’s learning progress and its ability to generalize unseen data. Significant differences between these curves may indicate overfitting, while similar and low values suggest a good balance between learning and generalization^31,32^. Figure 2 shows the validation loss across different models with varying numbers of input channels: three channels, six channels, and seven channels. The graph demonstrates that the model with three channels exhibits the highest validation loss, with significant fluctuations across the epochs. The model with six channels shows a more stable but still relatively high loss compared to the model with seven channels. The proposed model with seven channels achieved the lowest validation loss, showing consistent performance and minimal overfitting. The loss for this model decreases steadily and remains low across all epochs, showcasing its superior generalization capability compared to the other setting configurations.

### Confusion matrix results

**Fig. 3:**
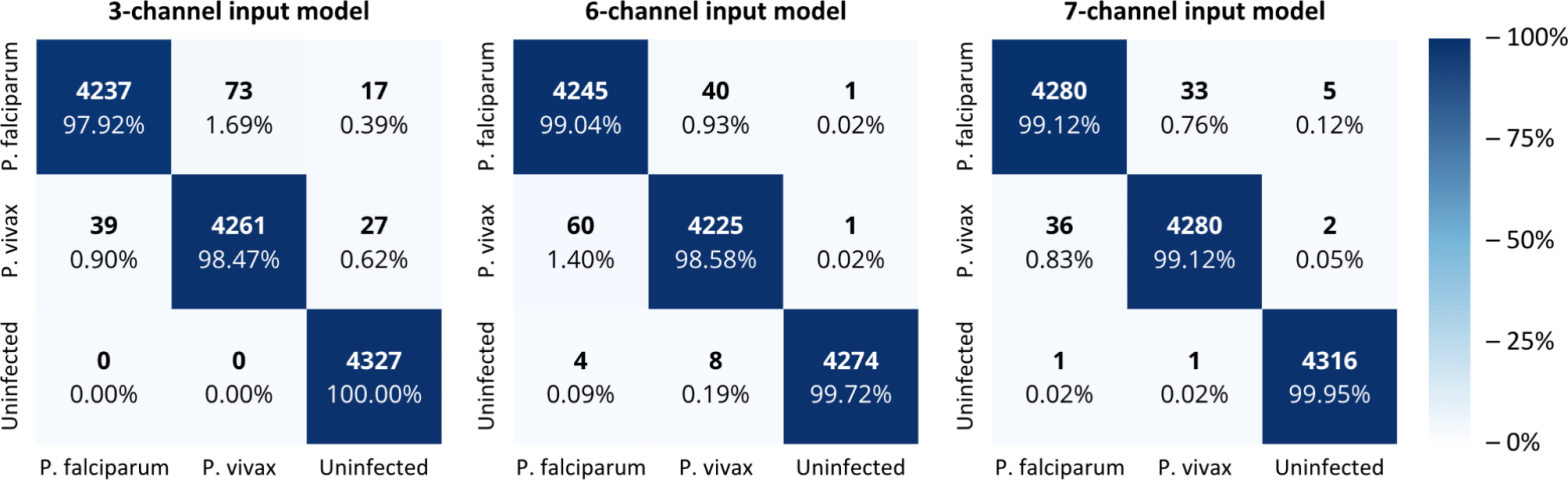
Confusion matrix results for proposed models by tensor input channels (Caracas, 2024). X-axis represents predicted labels and Y-axis represents true labels.

**Fig. 4:**
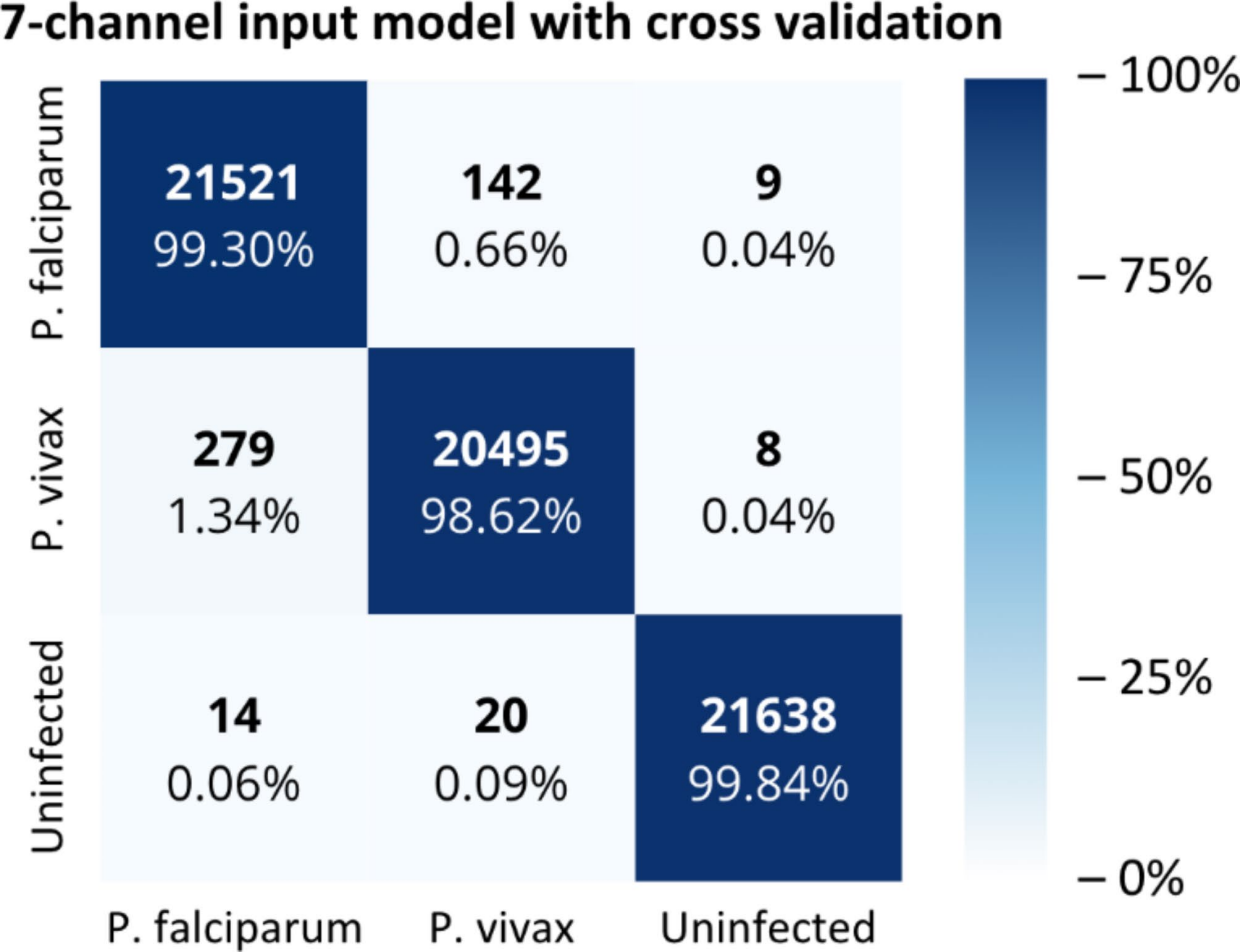
Confusion matrix results for seven-channel input model with cross validation (Caracas, 2024). X-axis represents predicted labels and Y-axis represents true labels.

To measure the model’s performance, we used the testing dataset after training and validation to review the model’s ability to generalize to new data. A multiclass confusion matrix was added to visualize the model’s performance throughout classification tasks by displaying the frequency of predictions for each class compared to the true classes^33–35^. In Figs. 3 and 4, the X-axis represents the predicted labels, while the Y-axis represents the true labels. In addition to counting each sample, we calculated the percentage of correctly classified instances relative to the total samples within each class, providing a detailed view of the model’s accuracy across various categories. The three-channel input model demonstrated exceptionally good performance with 12,825 true predictions out of 12,981. This particular model shows exceptional performance for the uninfected class, achieving 100% accuracy in its predictions. However, the other models surpassed its overall performance. The best model was the seven-channel input model with 12,876 true predictions out of 12,954 total predictions.

As a result, a confusion matrix was generated for the seven-channel input model using cross-validation (Fig. 4). This confusion matrix showed the results of five iterations, with each iteration rotating the folds according to the K-fold method, achieving 63,654 true predictions out of 64,126 total predictions (99.26% of accuracy). Species-specific accuracies were 99.3% for *P. falciparum*, 98.29% for *P. vivax*, and 99.92% for uninfected cells.

### Comparison with existing state-of-the-art models

Various computational techniques have been employed in the development of artificial intelligence models for malaria parasites identification^36–41^, with CNNs demonstrating superior performance. To assess the effectiveness of our proposed model, we compared it against several existing state-of-the-art approaches for malaria detection, as summarized in Table 2. Most prior studies have focused on binary classification—detecting the presence or absence of malaria parasites—without differentiating between species. In contrast, our model performs multiclass classification, distinguishing among *P. falciparum*, *P. vivax*, and uninfected cells. In terms of performance metrics, previous studies achieved high accuracy in binary classification. For example, Rajaraman et al.^42^ achieved 99.51% accuracy using the InceptionResNet-V2 architecture, while Mujahid et al.^43^ reported 97.57% accuracy with EfficientNet-B2. However, a critical metric in clinical settings—specificity, essential for accurate species identification—is often absent in many studies. Fuhad et al.^10^, for instance, achieved a specificity of 99.17% using thin smears. Notably, our model surpasses this with a specificity of 99.71%, relying solely on thick smears for classification. Few prior studies have incorporated advanced preprocessing techniques. For instance, Preethi et al.^25^ employed methods such as Gaussian filtering for noise reduction and brightness adjustments to enhance object detection, though their model’s performance remains significantly lower than ours. While Rajaraman et al.^42^ achieved a slightly higher precision (99.84%) compared to our model (99.26%), their study focused exclusively on binary classification using thin smears. In contrast, our model achieves similar accuracy (99.51%) while addressing the more complex task of multiclass classification on thick smears. Overall, our model demonstrates exceptional performance, achieving 99.51% accuracy, 99.26% precision, 99.26% recall, 99.63% specificity, and a 99.26% F1 score.

**Table 2:** Comparative performance of the proposed model against state-of-the-art approaches for malaria detection.

Method	Classification style	Accuracy	Precision	Recall	Specificity	F1 score
S. Rajaraman et al. (2019)^42^	Binary	99.51	99.84	−	−	99.50
K. M. Fuhad et al. (2020)^10^	Binary	99.23	98.92	99.52	99.17	99.22
A. Vijayalakshmi et al. (2020)^24^	Binary	93.13	−	−	−	−
S. Preethi et al. (2021)^25^	Multiclass	95.85	89.52	93.99	92.11	91.69
K. Hemachandran et al. (2023)^11^	Binary	96.73	96.80	96.88	−	96.74
M. Mujahid et al. (2024)^43^	Binary	97.57	96.59	98.62		97.55
Ours	Multiclass	99.51	99.26	99.26	99.63	99.26

These results underscore the robustness of our model, particularly in addressing challenges unique to clinical practice. Unlike thin smears, thick smears present greater difficulty in identifying the parasite species due to the absence of clearly visible infected individual RBCs^10,42^. This distinction is critical because clinical protocols for thick smear microscopy rely on white blood cells (WBCs) rather than RBCs for parasite detection. This is due to the preparation process of thick smears, where RBCs are lysed to concentrate parasites within a smaller volume, enhancing their visibility. As a result, thick smears are primarily used for detecting parasitemia, as they reveal the presence of parasites without preserving RBC morphology. This is essential because the lysis of RBCs allows clinicians to focus on identifying parasites among white blood cells, making thick smears highly effective for quantifying parasitemia but challenging for species differentiation. While previous models primarily focus on RBCs for parasite identification smear^41,43^, our model stands out by being trained on thick smears, where RBCs are lysed therefore parasite visibility is enhanced. This approach accurately identifies WBCs, which is essential in the standard protocol for diagnosing malaria in thick smears and also allows the calculation of parasitemia.

### Limitations and perspectives

This study’s dataset reflects a specific geographic region, which may restrict the generalizability of the findings to areas with differing levels of endemicity or a higher prevalence of species other than *P. vivax* and *P. falciparum*. To enhance the model’s global applicability, future efforts should focus on validating its performance using diverse datasets that encompass broader geographic regions and additional species, such as *P. ovale* and *P. malariae*. Another significant limitation is the absence of validation in actual clinical environments. While the current study utilized a well-annotated dataset obtained under controlled laboratory conditions, we are addressing this gap by creating a new dataset comprising images collected directly from our region. This new dataset will allow us to assess the model’s performance under real-world conditions, facilitate its integration into clinical workflows, and identify potential areas for refinement.

Although the primary focus of our study was on model development and performance evaluation, we acknowledge the critical role of interpretability in the adoption of deep learning models in clinical settings. Techniques such as Grad-CAM may provide insights into the model’s decision-making process, fostering trust and usability among medical professionals. Incorporating these interpretability techniques into future iterations of our work will help bridge the gap between technical advancements and practical applications in healthcare.

The model’s training required advanced computational resources; however, its implementation may be less resource-intensive. Future efforts should explore integrating pre-trained models into lightweight applications designed to operate on low-power devices, such as mobile phones or basic laptops. Such applications could utilize the pre-trained weights to perform real-time image analysis, enabling rapid diagnoses in resource-limited areas where trained microscopists may not be available. For effective clinical deployment, the model must be complemented with user-friendly interfaces and workflows tailored to clinical needs. For example, tools could be developed to allow users to upload thick smear images, such as photos captured directly from a microscope using a smartphone. Additional features, such as a cropping tool for highlighting suspected parasite structures, would enable the model to classify the image as parasite or non-parasite and, if applicable, identify the species. The current lack of such user-friendly workflows limits the model’s applicability in clinical settings. Addressing this gap will enhance the model’s practicality, align it with end-user requirements, and promote its adoption in resource-constrained environments.

## Methods

### Dataset compilation and annotation

**Fig. 5:**
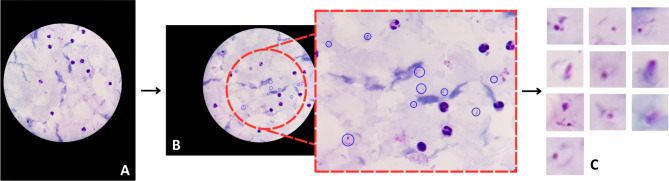
Workflow of microscopic to cellular image segmentation (Caracas, 2024). **A** Microscope level images, **B** parasites and cells detection over the microscope level images, **C** Cell level images.

This study utilized a dataset comprising microscopy images of Giemsa-stained thick blood smears collected at Chittagong Medical College Hospital, Bangladesh. The images were captured through the eyepiece of a microscope at 100X magnification using a smartphone camera. All images were saved in the RGB color space with a resolution of 3,024 × 4,032 pixels. The dataset was categorized into three subsets: smears infected with *P. falciparum*, smears infected with *P. vivax*, and smears from healthy individuals. In total, the dataset consisted of 5,941 thick smear images (at the microscope level) obtained from 350 patients, both infected and uninfected. The infection status of all patients was confirmed using the gold-standard method of thick and thin blood smear microscopy. These microscope-level images were subsequently processed to generate 190,399 individually labeled images cellular-level images (Table 3). Annotation of the dataset was performed by an expert microscopist from the Mahidol-Oxford Tropical Medicine Research Unit in Bangkok. The expert verified the presence of infection, identified the species, and manually annotated each image to label parasites and white blood cells. These annotated images were cataloged and archived in the National Library of Medicine^27^ (Fig. 5). The dataset has been previously validated, analyzed, and published by the same research group^9,44,45^.

**Table 3:** Summary of dataset composition and annotation.

Subset	No. of patients	No. of images	Annotated parasites	Annotated WBCs
*P. falciparum*	150	3,000	84,509	35,449
*P. vivax*	150	1,800	43,101	0
Uninfected	50	1,141	0	27,340
Total	350	5,941	127,610	62,789

### Dataset preprocessing

To ensure the model’s effectiveness and accuracy, we used high-quality data for the CNN. This fundamental step in ML models, known as data preprocessing, involved the following techniques:

#### Noise removal at the edges

**Fig. 6:**
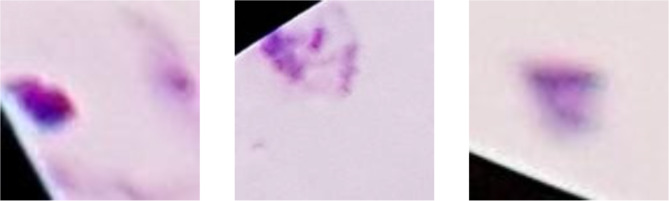
Instances of images from the dataset with noise (Caracas, 2024). Black edges near microscope borders illustrate noise affecting image preprocessing.

A considerable number of images contained black borders at the periphery, resulting from the microscope’s field of view (Fig. 6). These black borders were considered “noise” as they could negatively impact the neural network’s learning process by diverting attention from relevant features.

**Fig. 7:**
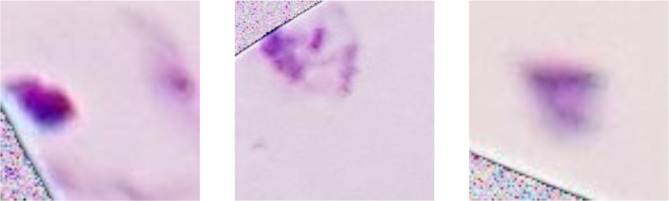
Replacement of noise in the images with white noise (Caracas, 2024). Black edge noise replaced with white noise to standardize image preprocessing.

We implemented the “White Noise” technique in these black areas to eliminate the noise. Although it may seem counterintuitive, White Noise helps in image processing by characterizing random pixel intensity variations uniformly across all frequencies. Each pixel has an independent value, with no predictable pattern for the model to learn from. We achieved this by setting a threshold to detect images where 10% or more of their pixels had an intensity of 40 or below on the grayscale. For these images, we applied White Noise by generating random pixel values based on a normal distribution using the mean and standard deviation of the colors present in the non-black areas (Fig. 7).

#### Enhancement of hidden features

**Fig. 8:**
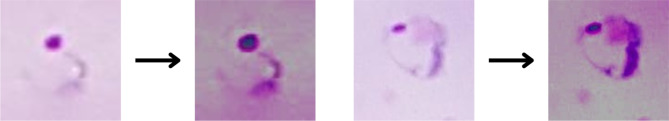
Instances of *P. falciparum* before and after the application of hidden feature enhancement (Caracas, 2024).

Certain images posed challenges in distinguishing between *P. falciparum* and *P. vivax* due to a lack of distinctive features. To address this, we applied filters to enhance subtle features that could aid the CNN in accurately detecting and classifying relevant patterns. We used three specific filters: contrast, saturation, and sharpness. These filters were implemented sequentially using the OpenCV library in Python, with experimentally determined parameters to best highlight the relevant features (Fig. 8). The resulting enhanced images contributed three additional RGB channels to the network’s input tensor. When combined with the original RGB channels, these enriched the dataset, improving the network’s ability to accurately identify and classify patterns.

#### Canny Edge Detection algorithm

**Fig. 9:**
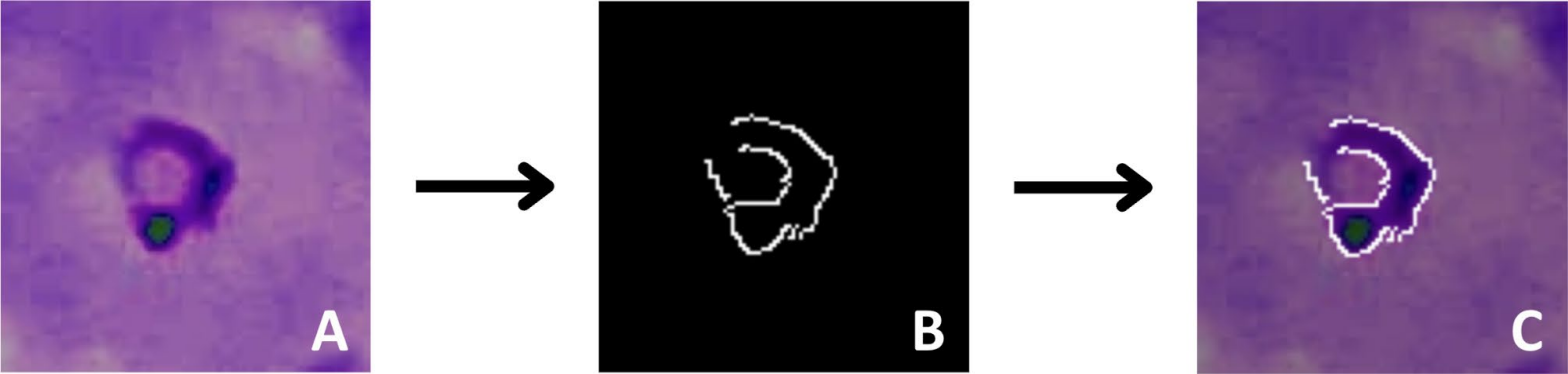
Workflow of Canny edge detection algorithm implementation (Caracas, 2024). **A** Enhanced image of *P. falciparum*. **B** Grayscale conversion as input for the Canny Edge Detection Algorithm. **C** Detected edge map superimposed as an additional channel over the enhanced image tensors.

To further emphasize key morphological features crucial for distinguishing *Plasmodium* species, we implemented the Canny Edge Detection algorithm using the OpenCV library. This algorithm is known for its ability to accurately detect edges. Since it requires grayscale input, we first converted the enhanced images to grayscale. The Canny function was then used to produce a binary image with white edges and a black background. This binary image was converted into a channel and integrated as an additional channel to the input tensor for the model, enhancing the CNN’s ability to differentiate the parasite’s morphological features (Fig. 9).

### Dataset balancing

Addressing class imbalance in the dataset was crucial for the model’s success. We employed the undersampling technique, reducing the number of samples in overrepresented classes to match those in underrepresented classes. This ensured balanced class representation during training. To minimize potential information loss from undersampling, we applied this technique within each fold during the K-fold cross-validation process. By adjusting the sample size in overrepresented classes for each fold, we maintained balanced representation throughout all stages of training.

### Custom model development

**Fig. 10:**
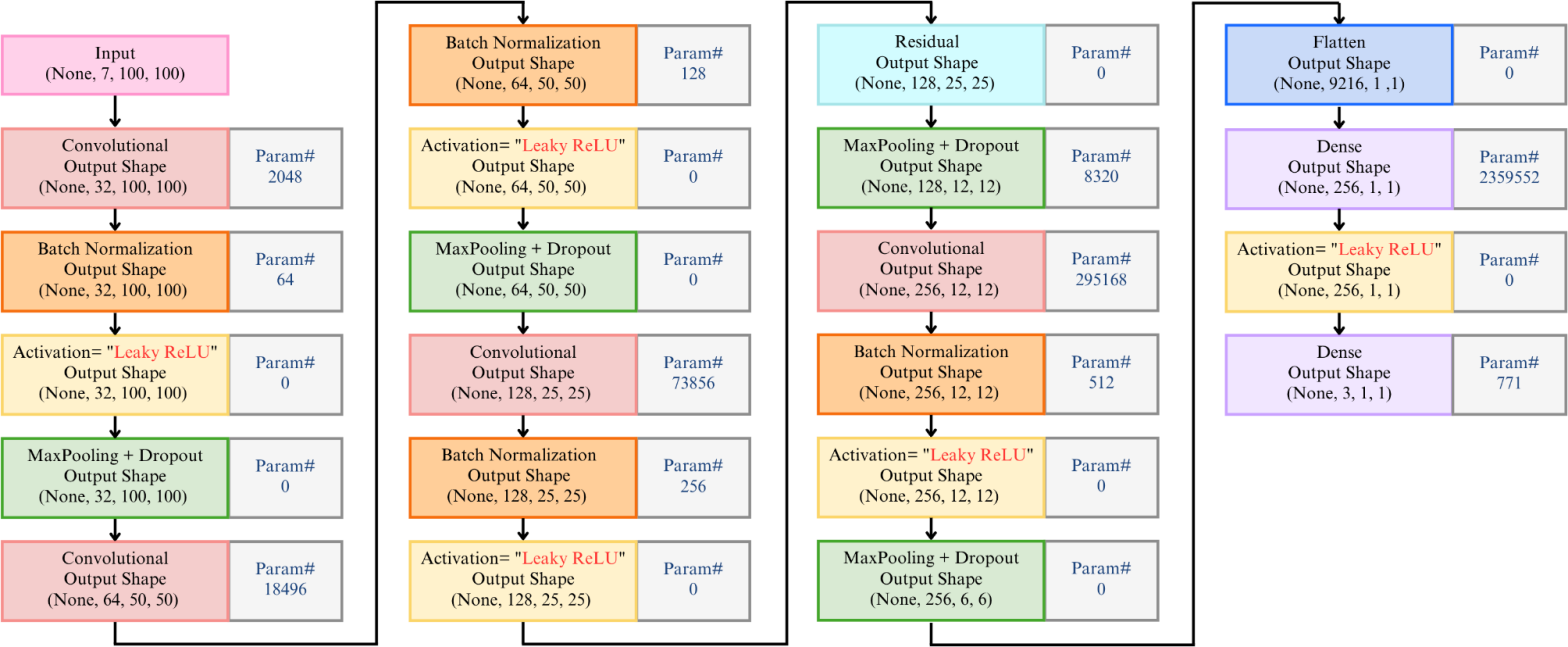
Architecture of proposed deep learning model (Caracas, 2024). Showing the sequence of convolutional layers with batch normalization, leaky ReLU activation and Max Pooling feature extraction, followed by fully connected layers for classification into three classes: *P. falciparum*, *P. vivax*, and uninfected.

We developed a custom CNN model composed of ten main layers, including four convolutional layers, four batch normalization layers with Leaky ReLU activation (alternating between the convolutional layers), and two fully connected layers. The convolutional layers use 3 × 3 filters, while the Max Pooling operations apply a 2 × 2 window with a stride that halves the dimensions of the feature maps after each pooling. Following the last Max Pooling layer, the features are flattened and processed through two fully connected layers, ultimately leading to a Softmax classifier. Figure 10 provides a complete diagram of our network.

The model’s performance was evaluated using various performance metrics (detailed in the “Performance measures” subsection), along with multiclass confusion matrices and training and validation loss functions. Both the confusion matrices and loss functions were fundamental in assessing classification accuracy and are explained in detail in the “Results and discussion” section.

### Performance measures

We evaluated the model’s performance using metrics such as accuracy, precision, recall, specificity, F1 score, and loss. These metrics were calculated based on values obtained from the confusion matrices, which include true positives (TP), true negatives (TN), false positives (FP), and false negatives (FN).

- Accuracy: Measures the proportion of correct predictions over the total number of predictions made.

$$\:\frac{TP+TN}{TP+TN+FP+FN}$$

- Precision: Measures the proportion of true positives relative to all positives identified by the model, reflecting how accurate the model is in identifying a case as positive.

$$\:\frac{TP}{TP+FP}$$

- Recall: Measures the proportion of true positives identified by the model relative to the total number of actual positive cases, reflecting how complete the model is in identifying all positive cases.

$$\:\frac{TP}{TP+FN}$$

- Specificity: Measures the proportion of true negatives identified by the model relative to the total number of actual negative cases, reflecting how effective the model is at ruling out non-positive cases.

$$\:\frac{TN}{TN+FP}$$

- F1 Score: The harmonic mean of recall and precision, particularly useful in scenarios where false positives and false negatives are equally costly.

$$\:2\times\:\frac{Precision\:\times\:\:Recall}{Precision\:+\:Recall}$$

Finally, the loss was calculated using a cross-entropy loss function implemented through PyTorch Lightning. This function evaluated the difference between predicted and actual class distributions, guiding the model’s learning process. In each training step, PyTorch Lightning computed the cross-entropy loss to adjust the model’s parameters, further optimizing performance by minimizing errors over time.

## Conclusion

The proposed model presents an efficient CNN-based approach for detecting parasitized cells and classifying *P. falciparum* and *P. vivax* in malaria-infected cells. Traditional microscopy methods face limitations in both accuracy and consistency due to operator dependency. Our model uniquely focuses on thick smear images, a task typically challenging for the human eye. By progressively incorporating advanced preprocessing techniques, the model’s performance improved significantly, achieving an accuracy of 0.9951, precision of 0.9926, recall of 0.9926, specificity of 0.9963, an F1 score of 0.9926, and a loss of 0.023. This approach not only simplifies the diagnostic process but also enhances accuracy and reliability, offering a powerful tool for resource-limited settings where traditional methods may be less effective. We are developing a database of images from remote areas to train our model for effective use in resource-limited environments, aiming to create a comprehensive diagnostic system where trained microscopists are unavailable.

## Data Availability

The dataset used and analyzed during the current study are available at: https://lhncbc.nlm.nih.gov/LHC-research/LHC-projects/image-processing/malaria-datasheet.html.
